# Supplementary material for: Phylogenomics of the Major Tropical Plant Family Annonaceae Using Targeted Enrichment of Nuclear Genes
Source: Front Plant Sci. 2019 Jan 9;9:1941. doi: 10.3389/fpls.2018.01941 (PMC6334231; doi:10.3389/fpls.2018.01941)
Supplement: Supplementary file 2 [file Data_Sheet_1.PDF]

Phylogenomics of the major tropical plant family Annonaceae using targeted enrichment of nuclear genes

Thomas L.P. Couvreur[1,*], Andrew J. Helmstetter[1], Erik J.M. Koenen[2], Kevin Bethune[1], Rita D. Brandão[3], Stefan Little[4], Hervé Sauquet[4,5], Roy H.J. Erkens[3]

1 IRD, UMR DIADE, Univ. Montpellier, Montpellier, France
2 Institute of Systematic Botany, University of Zurich, Zürich, Switzerland
3 Maastricht University, Maastricht Science Programme, P.O. Box 616, 6200 MD Maastricht, The Netherlands
4 Ecologie Systématique Evolution, Univ. Paris-Sud, CNRS, AgroParisTech, Université-Paris Saclay, 91400, Orsay, France
5 National Herbarium of New South Wales (NSW), Royal Botanic Gardens and Domain Trust, Sydney, Australia
*** thomas.couvreur@ird.fr**

Supplementary Information

(see next page)

Supplementary Table 1. Specimen details of taxa sampled for both Annonaceae and Piptostigmateae analyses

Subfamily	Tribe	Species	Collector	number	Country	INDEX	TAG	total reads	Mapped	% enrichment	10x coverage	mean depth
Ambavioideae		*Cleistopholis staudii*	Couvreur, T.L.P.	570	Gabon	I12	TAG79	1790158	402150	22	0,82	119,7
Ambavioideae		*Drepananthus ramuliflorus*	Sauquet, H.	167	Malaysia	I10	TAG45	2676926	150278	6	0,66	45,1
Ambavioideae		*Meiocarpidium olivieranum*	Couvreur, T.L.P.	920	Gabon	I10	TAG13	3950072	343879	9	0,80	104,3
Anaxagoreoideae		*Anaxagorea crassipetala*	Maas, P.J.M.	9408	Costa Rica	I10	TAG25	2648398	256748	10	0,67	76,9
Annonoideae	Annoneae	*Annona glabra*	Chatrou, L.W.	467	Peru	I10	TAG36	4328486	622387	14	0,83	190,7
Annonoideae	Annoneae	*Anonidium mannii*	Couvreur, T.L.P.	1053	Cameroon	I04	TAG36	1613002	679049	42	0,89	206,9
Annonoideae	Annoneae	*Boutiquea platypetala*	Couvreur, T.L.P.	386	Cameroon	I12	TAG71	3808288	1601840	42	0,91	484,8
Annonoideae	Annoneae	*Diclinanona calycina*	Pirie, M.D.	140	Peru	I10	TAG20	1650060	229310	14	0,73	68,9
Annonoideae	Annoneae	*Goniothalamus borneensis*	Onstein, R.E.	52	Malaysia	I10	TAG59	3491432	444488	13	0,80	137,9
Annonoideae	Annoneae	*Neostenanthera myristicifolia*	Couvreur, T.L.P.	841	Gabon	I12	TAG89	4268668	1826113	43	0,91	556,4
Annonoideae	Bocageeae	*Cymbopetalum costaricense*	Chatrou, L.W.	58	Costa Rica	I10	TAG30	2747196	533505	19	0,79	163,8
Annonoideae	Bocageeae	*Hornschuchia alba*	Maas, P.J.M.	8818	Brazil	I10	TAG32	7045436	765876	11	0,82	234,4
Annonoideae	Bocageeae	*Mkilua fragrans*	Couvreur, T.L.P.	25	Tanzania	I10	TAG43	5046552	1323802	26	0,84	411,0
Annonoideae	Bocageeae	*Porcelia venezuelensis*	Stergios, B.	20976	Venezuela	I10	TAG27	2737068	428786	16	0,78	131,7
Annonoideae	Duguetieae	*Duguetia staudtii*	Couvreur, T.L.P.	1014	Cameroon	I10	TAG17	2854728	440393	15	0,83	130,7
Annonoideae	Duguetieae	*Fusaea peruviana*	Pirie, M.D.	170	Peru	I10	TAG21	2747842	538885	20	0,86	163,8
Annonoideae	Duguetieae	*Letestudoxa bella*	Couvreur, T.L.P.	600	Gabon	I10	TAG10	2219212	476138	21	0,83	146,5
Annonoideae	Guatterieae	*Guatteria jefensis*	Maas, P.J.M.	9553	Panama	I10	TAG22	2703408	538079	20	0,85	164,2
Annonoideae	Monodoreae	*Asteranthe asterias*	Couvreur, T.L.P.	74	Tanzania	I10	TAG42	3817430	864889	23	0,86	266,5
Annonoideae	Monodoreae	*Isolona pleurocarpa*	Couvreur, T.L.P.	402	Cameroon	I10	TAG7	3246506	532526	16	0,83	161,8
Annonoideae	Monodoreae	*Mischogyne elliotianum*	Couvreur, T.L.P.	1033	Cameroon	I12	TAG99	3490880	1045328	30	0,88	315,5
Annonoideae	Monodoreae	*Monodora myristica*	Couvreur, T.L.P.	1031	Cameroon	I11	TAG7	710030	278735	39	0,55	68,2
Annonoideae	Monodoreae	*Sanrafaelia ruffonammari*	Couvreur, T.L.P.	6	Tanzania	I10	TAG44	3567626	719209	20	0,84	220,9
Annonoideae	Monodoreae	*Uvariastrum pierreannum*	Couvreur, T.L.P.	978	Cameroon	I12	TAG97	2739324	499467	18	0,87	150,1
Annonoideae	Monodoreae	*Uvariodendron molundense var. citrata*	Sosef, M.S.M.	2219	Gabon	I10	TAG8	3437590	609089	18	0,84	186,5
Annonoideae	Monodoreae	*Uvariopsis vanderystii*	Couvreur, T.L.P.	602	Gabon	I10	TAG11	4249308	393706	9	0,81	119,3
Annonoideae	Uvariaeae	*Dasymaschalon sp.*	Sauquet, H.	189	Malaysia	I10	TAG60	3964372	583594	15	0,83	178,3
Annonoideae	Uvariaeae	*Desmos sp.*	Couvreur, T.L.P.	839	Malaysia	I10	TAG53	5077150	907673	18	0,85	280,4
Annonoideae	Uvariaeae	*Fissistigma sp.*	Couvreur, T.L.P.	827	Malaysia	I10	TAG49	3482078	624289	18	0,83	191,6
Annonoideae	Uvariaeae	*Friesodielsia enghiana*	Couvreur, T.L.P.	1124	Gabon	I07	TAG50	1569122	475708	30	0,86	144,7
Annonoideae	Uvariaeae	*Friesodielsia excisa*	Sauquet, H.	177	Malaysia	I10	TAG54	4341290	593246	14	0,83	182,9
Annonoideae	Uvariaeae	*Monanthotaxis sp.*	Couvreur, T.L.P.	623	Cameroon	I06	TAG18	5319904	1756992	33	0,90	530,9
Annonoideae	Uvariaeae	*Monanthotaxis congoensis*	Couvreur, T.L.P.	1018	Cameroon	I12	TAG98	3885154	1221858	31	0,90	371,2
Annonoideae	Uvariaeae	*Monanthotaxis sp.*	Couvreur, T.L.P.	901	Gabon	I06	TAG32	5173764	1559966	30	0,90	478,0
Annonoideae	Uvariaeae	*Uvaria grandiflora*	Couvreur, T.L.P.	838	Malaysia	I10	TAG52	4284414	608782	14	0,84	188,0
Annonoideae	Xylopieae	*Artabotrys rufus*	Couvreur, T.L.P.	854	Gabon	I12	TAG90	2645832	726628	27	0,90	217,4
Annonoideae	Xylopieae	*Xylopia hypolampra*	Couvreur, T.L.P.	420	Cameroon	I12	TAG76	5301288	3465910	65	0,92	606,3
Annonoideae	Xylopieae	*Xylopia peruviana*	Chatrou, L.W.	483	Peru	I10	TAG37	3734334	617623	17	0,85	187,9
Malmeoideae	Bocageeae	*Trigynaea sp.*	Pirie, M.D.	97	Peru	I10	TAG16	2762520	341497	12	0,75	102,1
Malmeoideae	Malmeeae	*Cremastosperma oblongum*	Pirie, M.D.	7	Peru	I10	TAG15	2877928	442261	15	0,81	131,9
Malmeoideae	Malmeeae	*Ephedranthus parviflorus*	Prance, G.T.	19246	Brazil	I10	TAG33	2620664	577521	22	0,83	174,6
Malmeoideae	Malmeeae	*Klarobelia candida*	Chatrou, L.W.	124	Peru	I10	TAG31	3361894	568134	17	0,82	170,7
Malmeoideae	Malmeeae	*Mosannona vasquezii*	Chatrou, L.W.	226	Peru	I10	TAG34	3949068	519413	13	0,81	154,6
Malmeoideae	Malmeeae	*Onychopetalum periquino*	Chatrou, L.W.	439	Bolivia	I10	TAG35	3082266	679271	22	0,84	204,8
Malmeoideae	Malmeeae	*Oxandra asbeckii*	University of Guyana - Neotropical Botany	55	Guyana	I10	TAG38	2940064	442299	15	0,81	132,7
Malmeoideae	Malmeeae	*Pseudoxandra angustifolia*	Pirie, M.D.	139	Peru	I10	TAG19	2404874	471485	20	0,82	142,5
Malmeoideae	Malmeeae	*Ruizodendron ovale*	Chatrou, L.W.	453	Bolivia	I10	TAG28	3863196	1963829	51	0,85	282,0
Malmeoideae	Malmeeae	*Unonopsis spectabilis*	Pirie, M.D.	121	Peru	I10	TAG18	2573558	444856	17	0,81	133,5
Malmeoideae	Miliuseae	*Alphonsea kinabaluensis*	Couvreur, T.L.P.	820	Malaysia	I10	TAG61	3742072	490513	13	0,80	149,6
Malmeoideae	Miliuseae	*Desmopsis heteropetala*	Chatrou, L.W.	45	Costa Rica	I10	TAG29	2073504	174137	8	0,70	51,9
Malmeoideae	Miliuseae	*Meiogyne sp.*	Onstein, R.E.	43	Malaysia	I10	TAG46	4091290	707990	17	0,84	215,5
Malmeoideae	Miliuseae	*Monoon erianthoides*	Couvreur, T.L.P.	835	Malaysia	I10	TAG51	4289432	672068	16	0,83	207,4
Malmeoideae	Miliuseae	*Neo-uvaria acuminatissima*	Sauquet, H.	186	Malaysia	I10	TAG58	3551840	587049	17	0,82	178,9
Malmeoideae	Miliuseae	*Orophea sp.*	Onstein, R.E.	48	Malaysia	I10	TAG48	3692218	545374	15	0,82	167,1
Malmeoideae	Miliuseae	*Phaeanthus splendens*	Onstein, R.E.	73	Malaysia	I10	TAG57	3769494	550981	15	0,82	167,9
Malmeoideae	Miliuseae	*Polyalthia insignis*	Onstein, R.E.	47	Malaysia	I10	TAG47	3786194	652044	17	0,83	199,2
Malmeoideae	Miliuseae	*Popowia alata*	Sauquet, H.	180	Malaysia	I10	TAG55	3491736	506710	15	0,82	151,2
Malmeoideae	Miliuseae	Sageraea lanceolata	Onstein, R.E.	63	Malaysia	I10	TAG56	3854680	625144	16	0,83	190,9
Malmeoideae	Miliuseae	Sapranthus viridiflorus	Maas, P.J.M.	9402	Costa Rica	I10	TAG24	3548306	602362	17	0,82	181,2
Malmeoideae	Miliuseae	*Stenanona humilis*	Ishiki Ishihara, M.	2233	Mexico	I10	TAG26	2516866	215267	9	0,72	63,7
Malmeoideae	Monocarpieae	*Monocarpia kalimantanensis*	Couvreur, T.L.P.	831	Malaysia	I10	TAG50	3969066	761208	19	0,85	233,0
Malmeoideae	Piptostigmateae	*Annickia ambigua*	Wieringa, J.J.	6102	Gabon	I06	TAG12	3356760	1479731	44	0,90	447,8
Malmeoideae	Piptostigmateae	*Annickia ambigua*	Faye, A.	65	Republic of Congo	I06	TAG16	3783434	1561507	41	0,90	474,4
Malmeoideae	Piptostigmateae	*Annickia ambigua*	Couvreur, T.L.P.	788	Republic of Congo	I06	TAG28	4384950	1747534	40	0,91	530,4
Malmeoideae	Piptostigmateae	*Annickia chlorantha*	Couvreur, T.L.P.	414	Cameroon	I06	TAG7	4439712	1766979	40	0,90	373,0
Malmeoideae	Piptostigmateae	*Annickia kummerae*	Johnson, D.	1942	Tanzanie	I06	TAG50	4457360	2120713	48	0,91	631,5
Malmeoideae	Piptostigmateae	*Annickia kummerae*	Couvreur, T.L.P.	16	Tanzania	I06	TAG59	3694302	1721036	47	0,92	521,4
Malmeoideae	Piptostigmateae	*Annickia le testui*	Wieringa, J.J.	6369	Gabon	I06	TAG14	3328802	1380884	41	0,90	418,0
Malmeoideae	Piptostigmateae	*Annickia pilosa*	Couvreur, T.L.P.	859	Gabon	I06	TAG29	4166170	1716857	41	0,91	521,9
Malmeoideae	Piptostigmateae	*Annickia pilosa*	Couvreur, T.L.P.	866	Gabon	I06	TAG30	3822156	1563289	41	0,91	474,9
Malmeoideae	Piptostigmateae	*Annickia polycarpa*	Jongkind, C.C.H.	10892	Gabon	I06	TAG15	4482548	1721966	38	0,91	522,0
Malmeoideae	Piptostigmateae	*Annickia polycarpa*	Hawthorne, W.D.	122	Ghana	I06	TAG54	3137650	1174041	37	0,90	355,7
Malmeoideae	Piptostigmateae	*Annickia polycarpa*	Hawthorne, W.D.	195	Ghana	I06	TAG55	3003476	1134486	38	0,89	341,8
Malmeoideae	Piptostigmateae	*Brieya facsiculata*	Couvreur, T.L.P.	511	Cameroon	I06	TAG22	3215174	949340	30	0,90	283,0
Malmeoideae	Piptostigmateae	*Brieya facsiculata*	Couvreur, T.L.P.	645	Cameroon	I06	TAG23	3942350	1149663	29	0,91	344,0
Malmeoideae	Piptostigmateae	*Brieya fasciculata*	Couvreur, T.L.P.	578	Gabon	I06	TAG11	5169906	1570004	30	0,91	456,8
Malmeoideae	Piptostigmateae	*Brieya fasciculata*	Couvreur, T.L.P.	677	Cameroon	I06	TAG26	4095010	1123429	27	0,90	334,0
Malmeoideae	Piptostigmateae	*Greenwayodendron gabonicum*	Couvreur, T.L.P.	888	Gabon	I06	TAG31	4585004	1681497	37	0,91	494,0
Malmeoideae	Piptostigmateae	*Greenwayodendron gabonicum*	Couvreur, T.L.P.	1080	Gabon	I06	TAG42	4047080	1360155	34	0,90	399,1
Malmeoideae	Piptostigmateae	*Greenwayodendron gabonicum*	Wieringa, J.J.	8417	Gabon	I06	TAG51	5000256	1875494	38	0,91	551,3
Malmeoideae	Piptostigmateae	*Greenwayodendron glabrum*	Lissambou, B.J.	1807	Cameroun	I06	TAG65	4732552	1891374	40	0,91	564,1
Malmeoideae	Piptostigmateae	*Greenwayodendron glabrum*	Lissambou, B.J.	1745	Cameroun	I06	TAG68	5874848	2431999	41	0,91	726,9
Malmeoideae	Piptostigmateae	*Greenwayodendron litorale*	Wieringa, J.J.	8490	Gabon	I06	TAG52	5393460	2034896	38	0,92	614,9
Malmeoideae	Piptostigmateae	*Greenwayodendron litorale*	Breteler, F.	14481	Gabon	I06	TAG63	4444528	1824220	41	0,91	549,5
Malmeoideae	Piptostigmateae	*Greenwayodendron litorale*	de Wild, J.J.F.E.	11217	Gabon	I06	TAG64	3875460	1583631	41	0,91	477,5
Malmeoideae	Piptostigmateae	*Greenwayodendron oliveri*	Hawthorne, W.D.	WH213A_142	Ghana	I06	TAG60	3287894	1168579	36	0,89	343,2
Malmeoideae	Piptostigmateae	*Greenwayodendron oliveri*	Demenou, B.	2146	Liberia	I06	TAG61	3371818	1114977	33	0,89	329,0
Malmeoideae	Piptostigmateae	*Greenwayodendron oliveri*	Fofana, F.	120	Guinea Conakry	I06	TAG62	3981396	1303033	33	0,89	376,3
Malmeoideae	Piptostigmateae	*Greenwayodendron suaveolens*	Lissambou, B.J.	1748	Cameroun	I06	TAG66	3494224	1391552	40	0,90	414,2
Malmeoideae	Piptostigmateae	*Greenwayodendron suaveolens*	Lissambou, B.J.	2100	Cameroun	I06	TAG67	4313414	1679996	39	0,90	495,5
Malmeoideae	Piptostigmateae	*Greenwayodendron suaveolens*	Couvreur, T.L.P.	746	Gabon	I06	TAG70	3033312	1256624	41	0,91	536,4
Malmeoideae	Piptostigmateae	*Greenwayodendron suaveolens*	No Voucher		Gabon	I08	TAG13	1357304	385610	28	0,85	114,6
Malmeoideae	Piptostigmateae	*Greenwayodendron suaveolens*	No Voucher		Cameroon	I08	TAG21	1624856	480131	30	0,86	143,6
Malmeoideae	Piptostigmateae	*Greenwayodendron suaveolens*	No Voucher		Cameroon	I08	TAG26	1566684	451046	29	0,86	134,0
Malmeoideae	Piptostigmateae	*Greenwayodendron usambaricum*	Johnson, D.	1943	Tanzanie	I06	TAG49	1531392	677192	44	0,87	198,9
Malmeoideae	Piptostigmateae	*Mwasumbia alba*	Couvreur, T.L.P.	75	Tanzania	I06	TAG58	3669564	1215821	33	0,90	365,4
Malmeoideae	Piptostigmateae	*Piptostigma calophyllum*	Ghogue, J.P.	3000	Cameroon	I06	TAG35	3823320	1106504	29	0,90	324,9
Malmeoideae	Piptostigmateae	*Piptostigma cf mayndongtsaeanum*	Couvreur, T.L.P.	672	Cameroon	I06	TAG25	2357088	672009	29	0,88	196,2
Malmeoideae	Piptostigmateae	*Piptostigma goslineanum*	Couvreur, T.L.P.	983	Cameroon	I06	TAG34	3157428	860698	27	0,90	254,7
Malmeoideae	Piptostigmateae	*Piptostigma goslineanum*	Couvreur, T.L.P.	1017	Cameroon	I06	TAG36	3459784	900033	26	0,90	266,5
Malmeoideae	Piptostigmateae	*Piptostigma goslineanum*	Couvreur, T.L.P.	1030	Cameroon	I06	TAG37	3591846	909422	25	0,90	270,1
Malmeoideae	Piptostigmateae	*Piptostigma goslineanum*	Etuge, M.	2698	Cameroon	I06	TAG40	2236456	693480	31	0,88	199,8
Malmeoideae	Piptostigmateae	*Piptostigma goslineanum*	Couvreur, T.L.P.	1047	Cameroon	I06	TAG48	2804144	765386	27	0,89	225,9
Malmeoideae	Piptostigmateae	*Piptostigma macranthum*	Couvreur, T.L.P.	1134	Gabon	I06	TAG45	2624380	701187	27	0,89	207,9
Malmeoideae	Piptostigmateae	*Piptostigma macranthum*	Couvreur, T.L.P.	1135	Gabon	I06	TAG46	2780578	730606	26	0,89	216,6
Malmeoideae	Piptostigmateae	*Piptostigma macrophyllum*	Couvreur, T.L.P.	1034	Cameroon	I06	TAG38	2913116	801176	28	0,89	237,2
Malmeoideae	Piptostigmateae	*Piptostigma macrophyllum*	Parmentier I.	4980	Cameroun	I06	TAG56	1818166	528568	29	0,87	153,9
Malmeoideae	Piptostigmateae	*Piptostigma macrophyllum*	Couvreur, T.L.P.	518	Cameroon	I10	TAG12	2920742	257493	9	0,77	76,9
Malmeoideae	Piptostigmateae	*Piptostigma mortehanii*	Couvreur, T.L.P.	667	Cameroon	I06	TAG69	3766478	1159406	31	0,90	333,5
Malmeoideae	Piptostigmateae	*Piptostigma mortehanii*	Couvreur, T.L.P.	436	Cameroon	I06	TAG8	3248792	1073889	33	0,89	307,1
Malmeoideae	Piptostigmateae	*Piptostigma multinervium*	Couvreur, T.L.P.	616	Cameroon	I06	TAG17	3056936	835291	27	0,89	247,4
Malmeoideae	Piptostigmateae	*Piptostigma multinervium*	Couvreur, T.L.P.	637	Cameroon	I06	TAG21	3140378	1003777	32	0,93	298,3
Malmeoideae	Piptostigmateae	*Piptostigma multinervium*	Couvreur, T.L.P.	649	Cameroon	I06	TAG24	2088652	625754	30	0,88	183,3
Malmeoideae	Piptostigmateae	*Piptostigma oyemense*	Couvreur, T.L.P.	917	Gabon	I06	TAG33	2891216	861801	30	0,89	253,0
Malmeoideae	Piptostigmateae	*Piptostigma oyemense*	Couvreur, T.L.P.	1139	Gabon	I06	TAG47	3002840	845897	28	0,89	249,7
Malmeoideae	Piptostigmateae	*Piptostigma oyemense*	Couvreur, T.L.P.	523	Gabon	I06	TAG9	2832486	915356	32	0,88	264,6
Malmeoideae	Piptostigmateae	*Piptostigma pilosum*	Couvreur, T.L.P.	1070	Gabon	I06	TAG41	3296530	872132	26	0,89	258,3
Malmeoideae	Piptostigmateae	*Piptostigma pilosum*	Couvreur, T.L.P.	1103	Gabon	I06	TAG43	3289272	832337	25	0,90	247,3
Malmeoideae	Piptostigmateae	*Piptostigma sp.*	Wieringa, J.J.	6104	Gabon	I06	TAG13	2680916	809981	30	0,89	237,7
Malmeoideae	Piptostigmateae	*Piptostigma sp.*	Couvreur, T.L.P.	693	Cameroon	I06	TAG27	3479282	985769	28	0,90	289,9
Malmeoideae	Piptostigmateae	*Piptostigma submontanum*	Couvreur, T.L.P.	625	Cameroon	I06	TAG20	2762948	794248	29	0,89	231,2
Malmeoideae	Piptostigmateae	*Polyceratocarpus microtrichus*	Droissart, V.	1881	Cameroon	I06	TAG53	3390254	891160	26	0,90	268,2
Malmeoideae	Piptostigmateae	*Polyceratocarpus parviflorus*	Couvreur, T.L.P.	632	Cameroon	I06	TAG19	3753120	1017994	27	0,91	305,7
Malmeoideae	Piptostigmateae	*Polyceratocarpus parviflorus*	Couvreur, T.L.P.	1036	Cameroon	I06	TAG39	5016724	1203608	24	0,91	362,9
Malmeoideae	Piptostigmateae	*Polyceratocarpus pelligrinii*	Couvreur, T.L.P.	575	Gabon	I06	TAG10	3406604	944840	28	0,90	281,2
Malmeoideae	Piptostigmateae	*Sirdavidia solannona*	Couvreur, T.L.P.	1127	Gabon	I06	TAG44	4135966	1090730	26	0,91	328,4
Malmeoideae	Piptostigmateae	*Sirdavidia solannona*	Couvreur, T.L.P.	597	Gabon	I10	TAG9	3826504	422715	11	0,83	128,0

Protocol 1: DNA extraction

- **Step 1: For each individual, about 1-2 cm^2 of plant material (dried leaves) are put in a tube with a ceramic bead and grounded twice during 40 seconds at 6m/s using FastPrep-24tm 5G Instrument (MP BIOMEDICALS). Samples take then a powder-like appearance.**

- **Step 2: Samples are lysed during 3 hours at +65°C with 1mL of lysis buffer (100mM Tris, 25mM EDTA, 1.4M NaCl, 4 % MATAB, 1mM DTT) and 5µL of 1mg/mL proteinase K (Sigma-Aldrich, # P2308).**

- **Step 3: 10µL of 1mg/mL RNase (PROMEGA, # A797C) is added to each sample and incubation step at +37 °C for 1 hour is performed.**

- **Step 4: 1mL of chloroform:Isoamyl alcohol (24:1) is added to each sample and tubes are mixed vigorously before undergoing a centrifugation step at 6200g and +15°C during 20 minutes. Supernatant is removed and placed in another tube. Step 4 is done twice for a better purification of the DNAs.**

- **Step 5: 0.1 volume of sodium acetate (3M, pH=5) and 0.6 volumes of isopropyl alcohol thermostated to -20°C are added to 1 volume of supernatant. Tubes are mixed gently and a centrifugation step at 6200g and +4°C during 20 minutes is performed. Supernatant is discarded to keep the pellet at the bottom.**

- **Step 6: 1mL of ethyl alcohol at 70% is added to each sample. Tubes are mixed gently and a centrifugation step at 6200g and +4°C during 20 minutes is performed. Supernatant is discarded to keep the pellet at the bottom.**

- **Step 7: The pellet is dried a few minutes and then resuspended in 200µL of TE buffer (1X, pH=8). DNA concentration is checked on a NanoQuant Infinite M200 (Tecan Group Ltd, Seestresse 103 8708 Männedorf, Switzerland) and its quality is controlled on agarose gel electrophoresis.**

Protocol 2: Library preparation for multiplexed individuals follows the published protocol of Rohland & Reich (2012) with some modifications and the inclusion of 5 additional steps (5-9) for the enrichment procedure (biotinylated probes capture)

Library preparation (start)

- **Step 1:** For each individual, 100µL with around 3µg of total DNA are diluted in 0.65mL microtubes and sheared using Bioruptor Pico (Diagenode) to a targeted 300 bp DNA fragment size proceeding to 6 cycles of : 30 seconds "ON" + 30 seconds "OFF". Depending on the DNA degradation state, more or less cycles are applied. Sheared DNA size is checked on a QIAxcel 12-channel Capillary Electrophoresis System using the QIAxcel DNA screening kit (2400) (QIAGEN, Hilden, Germany, # 929004).

- **Step 2:** Sheared DNA is blunted and 5' phosphorylated using the NEBNext® End Repair Module kit (New England BioLabs® # E6050) by adding 5µL of 10X End Repair Reaction Buffer and 5µL of End Repair Enzyme Mix in a final volume of 50µL. The mix is incubated 30 minutes at room temperature (+22°C) and the reaction is stopped with Agencourt® AMPure® XP 1x clean-up step (Beckman Coulter Company, 500 Cummings Center, Suite 2450, Beverly, Massachusetts, # A63881). DNA concentration is checked on NanoQuant to ensure enough material is available for downstream steps.

- **Step 3 :** A total of 0.4 pmol of DNA fragments are ligated with 8pmol of MPE-P7 (TGACTGGAGTTCAGACGTGTGCTCTTCCGATCT), 8pmol of barcoded-P5-adapters (CTTTCCCTACACGACGCTCTTCCGATCT# 6-mer #) using hexamer barcodes observing an adapter:insert ratio of 20:1, 10 units T4 DNA ligase and 2µL of 10X Buffer T4 (Thermo Fisher Scientific, # EL0011 and # B69) in a final volume of 20µL and for 2.5 hours at +22°C followed by a heat inactivation step at +65°C for 10 minutes. A clean-up step is performed with 1x AMPure XP magnetic beads.

- **Step 4:** A nick fill-in step is performed using 16 units of Bst DNA polymerase (New England Biolabs, M0275), 3µL of 10X Thermo Pol Buffer and 1.5µL of 5mM dNTPs in a final volume of 30µL. The mix is incubated for 15 minutes at +37°C. A clean-up step is performed with 1x AMPure XP magnetic beads. DNA concentration is checked on NanoQuant to ensure enough material is available for downstream steps.

Enrichment steps

- **Step 5:** A pre-hybridization PCR is performed using 20µL of DNA, 25µL of KAPA HiFi HS Real-Time Master Mix (2X) (KAPA BIOSYSTEMS, # KM2702), 25pmol (2.5µL/10µM) of PreHyb-PE F primer (CTTTCCCTACACGACGCTCTTC) and 25pmol (2.5µL/10µM) of PreHyb-MPE R primer (TGACTGGAGTTCAGACGTGTG) following Rohland and Reich in a fi-

nal volume of 50µl and incubated in a LightCycler 480 Instrument II (Roche Molecular Systems, Inc.) for an initial 45 seconds at +98°C followed by 55 cycles of 15 seconds at +98°C, 30 seconds at +62°C and 30 seconds at +72°C ended by a single point fluorescence acquisition, with a final elongation of 1 minute at +72°C. PCR is monitored and stopped after optimal cycles are reached just before the plateau phase as recommended by manufacturer. In our case, this varied around 8-11 cycles, depending on the DNA quantity. A clean-up step is performed with 1x AMPure XP magnetic beads. DNA concentration is checked on NanoQuant to ensure enough material is available for downstream steps and fragment size is checked on the QIAxcel.

- Step 6 : Barcoded samples are equimolarly buked and in-solution hybridization capture is carried out using around 500ng to 1µg of total DNA in 7µL, previously denatured by heat during 5 minutes at +95°C, 5.5µL of biotinylated MyBaits probes, 2µL of 1% SDS, 12µL of 20X SSC, 1.3µL of 400ng/µL BSA, 0.5µL of 100µM MPE-P7 primer (TGACTGGAGTTCAGACGTGTGCTCTTC-CGATCT), 0.5µL of 100µM Univ Block P5+INOSINE primer (CTTTCCCTACACGACGCTCTTCCGATCTiiiiii) in a final volume of 30µL and incubated at +65°c for around 17-18 hours at 800rpm in a ThermoMixer® C (Eppendorf, Hamburg, # 5382000015).

- Step 7: Biotinylated probes annealed to the target complexes are then immobilized with 150µg of streptavidine-coated beads from the Streptavidin coupled Dynabeads C1 kit (Invitrogen, # 65001) previously washed and resuspended in 70µL with the 2X Binding Buffer supplied in the MyBaits kit. Immobilization is conducted in a 100µl final volume for 30 minutes at +65°C and 1200rpm in the ThermoMixer® C (Eppendorf, Hambourg, Germany). Samples are then placed on a magnet plate to remove the supernatant.

- Step 8: Beads are then subject to three washes with 500µl of Washing Buffer (WB 2.2 to prepare as recommended by the manufacturer), each wash including a 10 minutes incubation step at +65°C and 1200rpm in the ThermoMixer® C. Supernatant is then removed while samples are placed on a magnet plate as recommended by the manufacturer.

- Step 9: Beads are resuspended in 25µL of TT Buffer (10mM Tris-HCl, 0.05% Tween®-20) and incubated 5 minutes at +95°C to release hybridized DNA fragments. Samples are quickly placed on a magnet plate and the supernatant which contains the captured targeted DNA is retained.

Library preparation (continued)

- **Step 10: A Real-time post-hybridization PCR is undertaken to extend the adaptor sequence and enrich library fragments using 20μL of DNA, 25μL of 2X KAPA HiFi HS Real-Time Master Mix (KAPA BIOSYSTEMS, # KM2702), 25pmol (2.5μL/10μM) of Sol-PE-PCR F primer (AATGATACGGCGACCACCGAGATC-TACACTCTTTCCCTACACGACGCTCTTC) and 25pmol (2.5μL/10μM) of Sol-MPE-IND# R (CAAGCAGAAGACGGCATACGAGAT# 6-mer# GTGACTGGAGTTCAGACGTGT) following Rohland and Reich (2012) in a final volume of 50μl and incubated in a LightCycler® 480 Instrument II (Roche Molecular Systems, Inc.) for an initial 45 seconds at +98°C followed by 55cycles of 15 seconds at +98°C, 30 seconds at +62°C and 30 seconds at +72°C ended by a single point fluorescence acquisition, with a final elongation of 1 minute at +72°C. PCR is monitored and stopped after optimal cycles are reached just before the plateau phase as recommended by manufacturer. In our case, this varied around 14-16 cycles depending on the DNA quantity after capture. The amplicons are then purified by adding and mixing 1x volume of AMPure XP magnetic beads to the PCR products. After 5 minutes of incubation at room temperature, supernatant is removed and two 70% ethanol-washes are done. Air dried beads are resuspended in 25μl of DNase/RNase free water. Amplicon sizes are checked on the QIAxcel. DNA quantification was done using NanoQuant.**

Barcoded libraries were then sequenced on a HiSeq 2000 platform, with a flowcell plot of 18 pmoles and 1% PhiX.
